# Validierung des Geriatrie-Checks zur Identifikation geriatrischer Patienten in der Notaufnahme

**DOI:** 10.1007/s00391-020-01699-1

**Published:** 2020-02-28

**Authors:** Tobias Gerhard, Kristina Mayer, Ulrike Braisch, Dhayana Dallmeier, Michael Jamour, Jochen Klaus, Thomas Seufferlein, Michael Denkinger

**Affiliations:** 1 Geriatrische Forschung der Universität Ulm, AGAPLESION Bethesda Ulm, Zollernring 26, 89073 Ulm, Deutschland; 2 Geriatrisches Zentrum Ulm/Alb-Donau, Universitätsklinikum Ulm, Ulm, Deutschland; 3 Institut für Epidemiologie und Medizinische Biometrie, Universität Ulm, Ulm, Deutschland; 4 Geriatrische Rehabilitationsklinik Ehingen, Ehingen, Deutschland; 5 Klinik für Innere Medizin I, Universitätsklinikum Ulm, Ulm, Deutschland

**Keywords:** Geriatrie-Check, Identifikation geriatrischer Patienten, „Identification of Seniors at Risk“, Validierung, Notaufnahme, Geriatrisches Assessment, Screening, Geriatrie-Check, Identification of geriatric patients, Identification of Seniors at Risk, Validation, Emergency department, Geriatric Assessment, Screening

## Abstract

**Hintergrund:**

Der Geriatrie-Check wurde im Rahmen des Geriatriekonzept Baden-Württemberg zur Identifikation geriatrischer Patienten in der Notaufnahme entwickelt.

**Ziel:**

Bestimmung der konvergenten und prädiktiven Validität des Geriatrie-Checks zu Identifikation und Verlaufsprädiktion geriatrischer Patienten in der Notaufnahme.

**Material und Methoden:**

Prospektive Kohortenstudie zwischen November 2015 und April 2016 mit 146 Patienten, älter als 70 Jahre, der internistischen Notaufnahme der Uniklinik Ulm. Getrennte Erhebung durch Ärzte und Pflegende: Identification of Seniors at Risk (ISAR), Geriatrie-Check, weitere kognitive und funktionelle Assessments und als Endpunkte: Veränderung von Pflegestufe, Barthel-Index, Wohnform.

**Ergebnisse:**

Der ISAR klassifizierte *n* =117 Patienten als geriatrisch, der Geriatrie-Check *n* =107. Die Übereinstimmung betrug 78,1 %. Mit dem ISAR als Goldstandard zeigte der Geriatrie-Check eine Sensitivität von 82,0 % und eine Spezifität von 62,1 %. Der positiv- bzw. negativ-prädiktive Wert lag bei 89,7 % bzw. 46,1 %. Mit dem ISAR als Goldstandard war die Einschätzung der Pflege präziser als die der Ärzte überlegen (Sensitivität 70,5 % vs. 58 %; Spezifität 88,9 % vs. 83,3 %). Die prädiktive Validität 5 Monate nach Aufnahme bezüglich oben genannter Endpunkte war am besten für die Einschätzung durch Pflege und Ärzte (insbesondere die Spezifität). Beide Tests waren sehr sensitiv, aber wenig spezifisch.

**Diskussion:**

Der Geriatrie-Check ist dem ISAR vergleichbar. Die konvergente Validität unterscheidet sich nur wenig. Beide, ISAR und Geriatrie-Check, sind etwas sensitiver als Ärzte und Pflege. Bezüglich der prädiktiven Validität sind Ärzte und Pflege den Scores überlegen. Ein Algorithmus aus ISAR oder Geriatrie-Check mit nachfolgender Einschätzung durch Arzt oder Pflege könnte sich für eine bedarfsgerechte Ressourcenallokation am besten eignen.

## Abstract (en)

**Background:**

The geriatric check was developed for identification of geriatric patients in emergency departments (ED) as part of the concept for geriatric care in Baden-Württemberg.

**Aim:**

Determination of convergent and predictive validity of the geriatric check with respect to identification and outcome prediction of geriatric patients in the ED.

**Material and methods:**

A prospective cohort study between November 2015 and April 2016 including 146 patients older than 70 years in the internal medicine ED of Ulm University Hospital. Separate assessment by physicians and nursing staff of the following: identification of seniors at risk (ISAR), geriatric check, additional cognitive and functional assessments and for outcome: change in care index, Barthel index, living arrangements.

**Results:**

The ISAR classified 117 patients as geriatric patients and the geriatric check 107. The correlation was 78.1%. With ISAR as the gold standard the geriatric check showed a sensitivity of 82.0% and a specificity of 62.1%. The positive and negative predictive values were 89.7% and 46.1%, respectively. The identification by simple estimation was better for nurses than for doctors (sensitivity 70.5% vs. 58%, specificity 88.9% vs. 83.3%). The predictive validity 5 months after admission with respect to the abovementioned outcome parameters was best for nurses and doctors (especially regarding specificity). Both tests were very sensitive but not very specific.

**Discussion:**

The geriatric check is comparable to the ISAR. The convergent validity showed little difference. Both the ISAR and geriatric check were slightly more sensitive than doctors and nurses. Regarding predictive validity, doctors and nurses were superior to both scores. An algorithm starting with ISAR or geriatric check and followed by an estimation by doctors or nurses could be most suitable for optimal resource allocation.

## Hintergrund und Studienziel

Nach aktuellen Berechnungen des Statistischen Bundesamts werden bis 2060 allein in Deutschland 33 % der Bevölkerung ein Alter von 65 Jahren und höher erreicht haben [18]. Nach übereinstimmenden Definitionen mehrerer Fachgesellschaften kennzeichnet sich der geriatrische Patient durch ein höheres Lebensalter (überwiegend 70 Jahre oder älter) in Verbindung mit dem Vorhandensein einer geriatrietypischen Multimorbidität [6, 23]. Ältere Patienten in der Notaufnahme gelten als Hochrisikopatienten mit einer prolongierten Aufenthaltsdauer und einer höheren Wahrscheinlichkeit, längerfristig hospitalisiert bzw. institutionalisiert zu werden [3]. Die Wahrscheinlichkeit, innerhalb der ersten 3 Monate nach einem ungeplanten Aufenthalt in der Notaufnahme zu versterben, liegt bei dieser Patientengruppe bei ca. 10 %, das Risiko für einen funktionellen Abbau mit Autonomieverlust und Pflegebedürftigkeit bei ca. 20 % [1, 17]. Eine möglichst frühzeitige Identifikation geriatrischer Patienten im Rahmen des Erstkontakts mit dem Gesundheitssystem ist demnach geboten [17].

Das aktuell weltweit am häufigsten eingesetzte Screeninginstrument ist der Identification of Seniors at Risk (ISAR), welcher 2012 in einem gemeinsamen Positionspapier der 3 Gesellschaften Bundesverband Geriatrie (BVG) e. V., Deutsche Gesellschaft für Gerontologie und Geriatrie (DGGG) e. V. und Deutsche Gesellschaft für Geriatrie (DGG) e. V. als das zum damaligen Zeitpunkt am besten validierte „screening tool“ zur Identifikation geriatrischer Patienten benannt wurde [24]. Als weiteres Screeninginstrument liegt der „Geriatrie-Check“ vor [2]. Dieser wurde im Rahmen des „Geriatriekonzept Baden-Württemberg“ bereits 2013 sowohl zur Identifikation eines geriatrischen Patienten u. a. in der Notaufnahme als auch zur Diskriminierung eines daraus resultierenden, geriatrischen Behandlungsbedarfs entwickelt. Bislang liegen nur wenige Daten zu Anwendbarkeit und Aussagefähigkeit des Geriatrie-Checks vor. In einer Validierungsstudie an einer Kohorte neurologischer Patienten zeigte sich der Geriatrie-Check als praktikables Instrument zur Identifikation geriatrischer Patienten mit einer guten Korrelation zu klassischen „assessment tools“ [7].

Ziel dieser Studie war deshalb die Beurteilung der konvergenten und prädiktiven Validität des Geriatrie-Checks, verglichen zum ISAR-Screening als bisher verwendetem Goldstandard in der Notaufnahme, sowie zusätzlich zur klinischen Einschätzung des medizinischen Personals.

## Studiendesign und Methoden

Um die Aussagekraft und Anwendbarkeit des Geriatrie-Checks in der Notaufnahme beurteilen zu können, wurde eine prospektive Kohortenstudie mit Patienten der internistischen Notaufnahme des Universitätsklinikums Ulm durchgeführt.

### Teilnehmer, Ein- und Ausschlusskriterien

Im Zeitraum vom November 2015 bis April 2016 wurden *n* =146 Patienten rekrutiert, welche in der internistischen Notaufnahme des Universitätsklinikums Ulm behandelt wurden. Einschlusskriterien waren 70 Jahre und älter, Aufnahme über die internistische Notaufnahme am selben Tag der Studienrekrutierung und Einwilligung zur Studienteilnahme. War eine Demenz bekannt oder bestand der klinische Verdacht, erfolgte die Studienteilnahme nur nach Zustimmung durch die nächste Bezugsperson oder einen Betreuungsbevollmächtigten. Als Ausschlusskriterium zählte demnach nur das fehlende Einverständnis.

### Erhebung und Studiendesign

Die Datenerhebung erfolgte überwiegend tagsüber an Wochentagen und rein zufällig anhand einer jeweils aktuellen Belegungsliste der Notaufnahme konsekutiv durch 2 Medizinstudenten im klinischen Abschnitt des Medizinstudiums als unabhängige Rater. Bei Einwilligung wurden Daten zu folgenden 3 Zeitpunkten erhoben.

Am Tag der Aufnahme wurde Folgendes erfasst:

- Geriatrie-Check und ISAR,
- klinische Einschätzung durch den behandelnden Arzt und die betreuende Pflegekraft als dichotome Bewertung (geriatrischer Patient? Ja/nein). Die Ärzte oder Pflegekräfte wurden zuvor nicht bezüglich der Definition geriatrischer Patienten geschult oder speziell fortgebildet.

Am Tag 2 nach Aufnahme wurden zusätzlich erfasst:

- Barthel-Index [13],
- Informant Questionnaire on Cognitive Decline (IQCODE) Score [9],
- Nutritional Risk Screening (NRS) [11],
- Kurzer Mentaler Test für Delir (KMT) [19],
- Geriatric Depression Scale – 4 Items (GDS-4) [12, 26].

Bei der Nachuntersuchung 3 bis maximal 5 Monate nach Einweisung wurden erfasst:

- Veränderung der Wohnsituation und Pflegestufe,
- Barthel-Index [13],
- IQCODE-Score [9],
- GDS‑4 [12, 26],
- Behandlungsverlauf und Kontakt mit weiterer geriatrischer Versorgung.

Der Drittkontakt im Anschluss an den Klinikaufenthalt fand mit den Studienteilnehmern sowie nach Möglichkeit (Zustimmung der Hausärzte zur Validierung der Daten, *n* =92) auch mit deren Hausärzten 3 bis 5 Monate nach der Versorgung in der Notaufnahme statt.

### Geriatrie-Check

**Figure 1:**
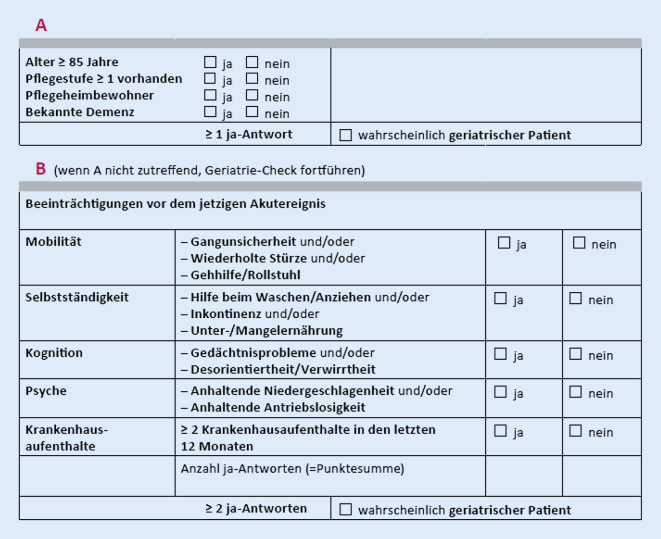

Der Geriatrie-Check teilt sich in 2 Teilbereiche auf (Abb. 1). Im Teil A wird das Vorliegen objektiver Kriterien geprüft, während Teilbereich B die subjektive Einschätzung des Patienten oder von dessen Bezugsperson erhebt. Patienten sind dem Geriatrie-Check nach als wahrscheinlich geriatrisch einzuschätzen, wenn bereits im Teilbereich A ein Punkt erzielt wird. In diesem Fall kann der Test beendet werden, wodurch eine Zeitersparnis im klinischen Alltag erwartet wird. Wenn nicht, wird Teil B durchgeführt, welcher den prämorbiden Gesundheitszustand des Patienten direkt vor der Akuteinweisung in das Krankenhaus untersucht. Für diesen Teilbereich gilt, dass bei mindestens 2 positiv bewerteten Subkategorien von einem wahrscheinlich geriatrischen Patienten auszugehen ist.

### Identification of Seniors at Risk

Beim 1999 entwickelten ISAR handelt es sich um einen vielfach validierten Selbstbeurteilungsfragebogen zur Identifikation geriatrischer Patienten in der Notaufnahme, welche infolge einer Akuterkrankung und der Krankenhausbehandlung einem erhöhten Risiko für negative Gesundheitsfolgen wie Mortalität, Institutionalisierung und Hospitalisierung sowie einem klinisch relevanten funktionellen Abbau ausgesetzt sind [14]. Der ISAR überprüft mittels eines einfachen dichotomen Aufbaus das Vorliegen geriatrietypischer Risikofaktoren, wie funktionelle Abhängigkeit in Form von erhöhtem prämorbidem Hilfsbedarf, einen aktuell verstärkten Bedarf an Unterstützung, den Zeitpunkt des letzten Klinikaufenthalts, eine subjektive kognitive Beeinträchtigung sowie das Vorliegen einer nichtkorrigierbaren Sehbehinderung und einer Multimedikation.

### Datenanalyse

Charakterisierung der Studienteilnehmer durch Bestimmung der absoluten und relativen Häufigkeit sowie der Lage- und Streuungsmaße. Die Überprüfung der Normalverteilung bei stetigen Variablen erfolgte mithilfe von Histogrammen und des Kolmogorow-Smirnow-Anpassungstests. Annähernd normal verteilte Variablen wurden mit Mittelwert und Standardabweichung (SD), nichtnormalverteile Variablen mit Median sowie erstem und drittem Quartil (Q1; Q3) beschrieben. Für den statistischen Vergleich zweier unabhängiger Gruppen wurden bei stetig und normal verteilten Variablen der *t*-Test, bei stetig und nicht normal verteilten Variablen der Wilcoxon-Mann-Whitney-U-Test und bei kategoriellen Variablen der Chi-Quadrat-Test durchgeführt.

Für die konvergente Validität und Diskriminierungsfähigkeit wurden der Geriatrie-Check, die klinische Einschätzung durch den behandelnden Arzt und durch die betreuende Pflegekraft mit dem ISAR als Goldstandard verglichen. Es wurde der Anteil der Übereinstimmung, die Sensitivität und Spezifität sowie der positive (PPW) und negative prädiktive Wert (NPW) berechnet.

Hinsichtlich der Bestimmung der prädiktiven Validität wurden vorab folgende Merkmale als primäre Endpunkte definiert: Verschlechterung des Barthel-Index (≥10 Punkte), der Wohnform (Zuhause ohne Hilfen →Zuhause mit Hilfen →betreutes Wohnen →Zuhause mit 24-h-Betreuung →Kurzzeitpflege und stationäres Pflegeheim →Krankenhaus) und der Pflegestufe. Im ersten Schritt wurden der Geriatrie-Check mit den primären Endpunkten anhand von Kreuztabellen verglichen und der Anteil der Übereinstimmung sowie die Sensitivität und Spezifität bestimmt.

Ebenso wurde versucht, anhand der Rückmeldungen der zuständigen Hausärzte, mittels direkter Auswertung der patientenspezifischen Behandlungsdaten und den Informationen aus den Entlassungsbriefen, die Patienten zu erfassen, welche einer geriatrischen Weiterversorgung zugeführt wurden.

Die statistischen Analysen wurden mit dem Statistikprogramm IBM Statistics SPSS (Version 24; IBM Corp., Armonk, NY, USA) durchgeführt. Alle statistischen Tests waren 2‑seitig, und *p* <0,05 wurde als statistisch signifikant definiert.

## Ergebnisse

### Charakteristika der Studienpopulation

Wie in Tab. 1 gezeigt, betrug das mediane Alter der Studienteilnehmer 79,8 Jahre, wobei das Verhältnis zwischen männlichen und weiblichen Teilnehmern nahezu ausgeglichen war. Von den 146 Studienteilnehmern wurden 107 Patienten (73,3 %) durch den Geriatrie-Check und 117 (80,1 %) durch ISAR als geriatrisch identifiziert. Die nach den Kriterien des Geriatrie-Checks als geriatrisch identifizierte Patienten waren, verglichen mit den nichtgeriatrischen Patienten im Durchschnitt älter, hatten bei Erstkontakt eine höhere Anzahl an Dauermedikation, einen niedrigeren Barthel-Index und einen höheren IQCODE-Score. Bei Aufnahme waren 14,0 %, 9,3 % und 2,8 % der als geriatrisch identifizierten Patienten jeweils in den Pflegestufen 1, 2 bzw. 3. Zudem zeigten sich statistisch signifikante Unterschiede zwischen nach Geriatrie-Check klassifizierten geriatrischen und nichtgeriatrischen Patienten, bezogen auf ein erhöhtes Risiko für Malnutrition (NRS) und Delir (KMT Delir).

**Table 1:** 

Variable	Patienten gesamt (*n* =146)	Geriatrie-Check		ISAR-Screening	
		Positiv (*n* =107)	Negativ (*n* =39)	Positiv (*n* =117)	Negativ (*n* =29)
Alter (Jahre), Median (Q1; Q3)	79,8 (77,0; 86,7)	81,0 (77,9; 88,1)	77,4 (74,4; 80,0)	80,6 (77,2; 86,8)	77,5 (75,0; 80,1)
Männer, *n* (%)	72 (49,3)	50 (46,7)	22 (56,4)	59 (50,4)	13 (44,8)
Anzahl, Verstorbene, *n* (%)	24 (16,4)	22 (20,6)	2 (5,1)	22 (18,8)	2 (6,9)
Barthel-Index vor Aufnahme, Median (Q1; Q3)	95 (80; 100)	95 (75; 100)	100 (100; 100)	95 (80; 100)	100 (100; 100)
Barthel-Index bei Drittkontakt, Median (Q1; Q3)	85 (50; 100) *n* =139	80 (45; 100) *n* =101	100 (90; 100) *n* =38	80 (45; 100) *n* =110	100 (95; 100) *n* =29
IQCODE-Score, Median (Q1; Q3)	3,3 (3,0; 3,6)	3,3 (3,1; 3,8)	3,1 (3,0; 3,3)	3,3 (3,1; 3,7)	3,1 (3,0; 3,4)
NRS positiv, *n* (%)	96 (65,8)	76 (71,0)	20 (51,3)	80 (68,4)	16 (55,2)
KMT Delir positiv, *n* (%)	88 (64,2)	70 (71,4)	18 (46,2)	74 (67,3)	14 (51,9)
GDS‑5 depressiv, *n* (%)	30 (20,5)	26 (24,3)	4 (10,3)	27 (23,1)	3 (10,3)
Leben im Griff^a^, *n* (%)	104 (71,2)	71 (68,2)	33 (84,6)	80 (68,4)	24 (82,8)
Institutionalisierung bei Aufnahme, *n* (%)	18 (12,3)	17 (15,9)	1 (2,6)	17 (14,5)	1 (3,4)
Pflegestufe vor Aufnahme, *n* (%)	28 (19,2)	28 (26,1)	0 (0,0)	27 (23,1)	1 (3,4)
Pflegestufe bei Drittkontakt, *n* (%)	50 (35,0) *n* =143	48 (46,2) *n* =104	2 (5,1) *n* =39	49 (42,9) *n* =114	1 (3,4) *n* =29
Anzahl, Dauermedikation, Median (Q1; Q3)	7,0 (5,0; 9,0)	7,0 (6,0; 10,0)	5,0 (3,0; 7,0)	7,0 (6,0; 10,0)	3,0 (2,0; 5,0)
Krankenhausaufenthalt (Tage), Median (Q1; Q3)	6,0 (2,5; 10,0)	6,5 (3,0; 11,0)	5,0 (2,0; 9,0)	7,0 (2,0; 11,0)	4,0 (3,0; 7,0)

Q1 =1. Quartil (25 %-Quantil), Q3 =3. Quartil (75 %-Quantil)

*IQCODE* Informant Questionnaire on Cognitive Decline, *NRS* Nutritional Risk Screening, *KMT* Kurzer Mentaler Test für Delir, *GDS* Geriatric Depression Scale – 4 Items

^a^Eigene Aussage

### Konvergente Validität

Verglichen mit dem ISAR als Goldstandard, konnten für den Geriatrie-Check eine höhere Sensitivität und eine schlechtere Spezifität als für die Einschätzung der Pflege sowie der Ärzte gezeigt werden. Zudem konnten diesbezüglich die Pflegekräfte für alle in Tab. 2 dargestellten Gütekriterien bessere Ergebnisse erzielen als die Ärzte.

**Table 2:** 

Gütekriterium	Geriatrie-Check (*n* =146) (%)	Arzt (*n* =105) (%)	Pflege (*n* =139) (%)
Übereinstimmung	78,1	63,8	74,1
Sensitivität	82,0	58,0	70,5
Spezifität	62,1	83,3	88,9
Positiver prädiktiver Wert	89,7	92,2	96,3
Negativer prädiktiver Wert	46,1	37,0	42,1

*n* Anzahl der ausgewerteten Patienten

### Prädiktive Validität

Es zeigte sich ein signifikanter Zusammenhang zwischen der Bewertung des Geriatrie-Checks und der Veränderung des Barthel-Index, wobei der Anteil an Patienten mit einer Verschlechterung im Barthel-Index unter den als geriatrisch identifizierten Personen höher war. Der Anteil der im Pflegeheim untergebrachten Patienten stieg von 10,3 % auf 21,2 % an. Der Anteil an Patienten mit einer Verschlechterung der Pflegestufe war bei den als geriatrisch identifizierten Patienten signifikant höher als bei den nichtgeriatrischen.

Eine Übersicht für den Geriatrie-Check, den ISAR, die Einschätzung des Arztes und der Pflegekraft, jeweils verglichen mit den vorab definierten, primären Endpunkten gibt Tab. 3. Hierbei zeigt der Geriatrie-Check bei weitgehend moderater bis guter Sensitivität einerseits eine geringfügige Unterlegenheit gegenüber dem ISAR-Screening, anderseits wird gleichzeitig die durchweg eingeschränkte Spezifität beider Assessment-Instrumente deutlich. Die klinische Einschätzung durch Pflege- oder ärztliches Personal war beträchtlich spezifischer über alle primären Endpunkte hinweg.

**Table 3:** 

Assessment		Verschlechterung des Barthel-Index	Verschlechterung der Wohnform	Verschlechterung der Pflegestufe
Geriatrie‐Check	Sensitivität	79,2 %	82,1 %	91,7 %
	Spezifität	39,7 %	36,0 %	36,8 %
	Signifikanz	0,022 (*n* =116)	0,086 (*n* =114)	0,019 (*n* =119)
ISAR‐Screening	Sensitivität	90,6 %	92,9 %	100,0 %
	Spezifität	34,9 %	29,1 %	28,4 %
	Signifikanz	<0,001 (*n* =116)	0,010 (*n* =114)	0,010 (*n* =119)
Einschätzung Arzt	Sensitivität	70,7 %	73,3 %	76,5 %
	Spezifität	80,9 %	64,8 %	64,4 %
	Signifikanz	<0,001 (*n* =88)	0,018 (*n* =86)	0,004 (*n* =90)
Einschätzung Pflege	Sensitivität	80,0 %	83,3 %	91,7 %
	Spezifität	72,1 %	58,8 %	58,9 %
	Signifikanz	<0,001 (*n* =111)	<0,001 (*n* =109)	<0,001 (*n* =114)

*n* Anzahl der ausgewerteten Patienten

### Kontakt zu anschließender geriatrischer Versorgung

Im Verlauf des Beobachtungszeitraums hatten insgesamt 22 Patienten Kontakt zu einer weiteren *akut*geriatrischen Versorgung. Davon waren 21 Patienten vom Geriatrie-Check und auch von den Pflegekräften als geriatrisch identifiziert worden. Hierbei handelt es sich um dieselben Patienten. Der ISAR hingegen wies allen 22 Patienten einen geriatrischen Status zu, die betreuenden Ärzte 16 von 19 erfassten Patienten (3 „missing values“). Bezüglich geriatrischer Rehabilitation fanden sich in den Entlassungsbriefen der Notfallambulanz keine Informationen. Die Hausarztrückmeldung war zu gering, um hier weitere Aussagen zu treffen.

## Diskussion

Der Geriatrie-Check des Landes Baden-Württemberg zeigt im Vergleich mit dem ISAR eine gute konvergente Validität. Pflege und Ärzte erzielen jeweils spezifischere Ergebnisse als der Geriatrie-Check selbst, sind jedoch weniger sensitiv bei der Identifizierung geriatrischen Patienten, wobei die Pflege insgesamt besser abschneidet als die Ärzte. Bei der Prüfung der prädiktiven Validität erreicht der Geriatrie-Check, bezogen auf die Endpunkte Verschlechterung des Barthel-Index, Verschlechterung der Wohnform sowie Verschlechterung einer Pflegestufe, ähnliche Ergebnisse. Bei durchweg moderater bis guter Sensitivität bleibt der ISAR dem Geriatrie-Check leicht überlegen. Beide Instrumente weisen jedoch eine eingeschränkte Spezifität auf. Innerhalb dieser Kategorie konnten die Pflegekräfte und v. a. die ärztliche Einschätzung deutlich bessere Resultate erzielen. Eine akutgeriatrische Nachversorgung erhielten nur etwa 18–20 % der über den Geriatrie-Check oder den ISAR identifizierten Patienten.

Zur Beurteilung der Diskriminationsfähigkeit des Geriatrie-Checks wurde der Endpunkt geriatrisch bzw. nichtgeriatrisch in der durchgeführten Studie anhand der Ergebnisse des ISAR bei einem Cut off (Trennwert) ≥2 Summenpunkten definiert (Goldstandard Cut off) [25]. In der Originalarbeit von McCusker et al. erzielte der ISAR unter Anwendung dieses Cut off lediglich eine mittelmäßige Sensitivität (72 %) bei einer gleichzeitig moderaten Spezifität (58 %) hinsichtlich des tatsächlichen Auftretens von negativen Gesundheitsfolgen wie Mortalität, Institutionalisierung, Hospitalisierung oder funktionellem Abbau während der ersten 6 Monate nach Entlassung. Bezogen auf die Identifikation von Frailty zeigte der ISAR mit demselben Cut off eine deutlich höhere Diskriminationsfähigkeit (AUC [Area Under the Curve] 0,92) und erreichte eine sehr gute Sensitivität (94 %), einhergehend mit einer jedoch ebenfalls nur moderaten Spezifität (63 %) [14, 21]. Der Geriatrie-Check geht dagegen im erweitertem Umfang auf typisch geriatrische Syndrome, wie Mobilität, Selbstständigkeit, Kognition und Psyche ein, identifiziert aber mit diesem Vorgehen eine vergleichbare Population wie der ISAR mit, wie in dieser Arbeit gezeigt, vergleichbaren psychometrischen Testmerkmalen.

Obwohl generell nicht bestritten wird, dass Screeninginstrumente auch in Notaufnahmen dazu beitragen könnten, Patienten bezüglich weiterer notwendiger Versorgungsformen zu identifizieren, wird diesen bislang eine unzureichende prognostische Genauigkeit bescheinigt [4]. Die Kerninhalte der zur Verfügung stehenden Instrumente ähneln sich sehr und finden sich auch in den erstmals 2016 von den deutschsprachigen geriatrischen Fachgesellschaften zusammen mit der Deutschen Gesellschaft für interdisziplinäre Notfall- und Akutmedizin (DGINA) veröffentlichten Qualitätsindikatoren für die Versorgung älterer Patienten in der Notaufnahme wieder [23]. Zu den Indikatoren, welche mit der höchsten Relevanz bewertet wurden, zählen neben dem obligaten Ausschluss eines Delirs v. a. die Identifizierung des geriatrischen Patienten, die systematische Erfassung von Vulnerabilität und Risikofaktoren älterer Patienten wie auch die Diskriminierung unterschiedlichen Handlungsbedarfs [23].

Betrachtet man relevante geriatrische Endpunkte (Barthel-Index, Wohnform, Institutionalisierung und Pflegestufe) erweist sich der Geriatrie-Check gegenüber der klinischen Einschätzung durch den Arzt bzw. die Pflegekraft als sensitiveres, aber nicht spezifisches Screeninginstrument. Der ISAR ist dem Geriatrie-Check bezüglich Sensitivität sogar überlegen. Beide Tests leiden allerdings unter einer niedrigen Spezifität. Hier sind Pflege und v. a. die ärztliche Einschätzung, welche ohne gesonderte geriatrische Schulung erfolgte, über alle Endpunkte hinweg überlegen. Konsekutiv wäre dementsprechend eine Kombination aus Screeninginstrument und ärztlicher bzw. pflegerischer Einschätzung zu empfehlen. Es wundert deshalb nicht, dass die Anwendung der Screeninginstrumente und zusätzlich eine spezialisierte klinische Weiterbildung der Ärzte und Pflegekräfte gefordert werden [1, 20, 22]. Nur wenige Arbeiten konnten jedoch einen Vorteil im weiteren klinischen Verlauf der Patienten durch Screeningverfahren zeigen [10]. Dass gegenwärtig dennoch mehrere Instrumente entwickelt und eingesetzt werden, ist u. a. durch eine Schärfung des Bewusstseins für geriatriespezifische Aspekte innerhalb der Notaufnahmen zu erklären [8]. So erwies sich die Einleitung einer geriatrischen Intervention auf Grundlage der ISAR-Ergebnisse, innerhalb eines 2‑stufigen Verfahrens, als effektive Methode zur Reduktion der Mortalität und eines funktionellen Abbaus, ohne dabei relevant höhere Kosten zu verursachen [15].

Ob mögliche Interventionspotenziale bereits in der Notaufnahme kursorisch mitidentifiziert werden sollten, bliebe ebenfalls zu prüfen. Anderenfalls müsste bei Auffälligkeit im Screening ein geriatrisches Assessment gefordert werden, mit der etwaigen Konsequenz einer notwendigen Verlegung in eine geriatrische Einrichtung. Denkbar wäre jedoch auch, orientierend weitere mögliche Interventionspotenziale bereits in der Notaufnahme zu identifizieren und damit eine sinnvolle Allokation weiterer Ressourcen zu erreichen. Hierunter kann man sich eine gezielte Weiterversorgung in einer Fachabteilung wie z. B. der Kardiologie oder Chirurgie, einer Akutgeriatrie, ambulanter oder stationärer Rehageriatrie oder auch die direkte Entlassung nach Hause mit folgender Vorstellung beim Hausarzt und/oder in der geriatrischen Institutsambulanz vorstellen. Für die Weiterentwicklung des Geriatrie-Checks könnte hier der sog. SEISAR ein interessantes Instrument zum ergänzenden Assessment in der Notaufnahme darstellen [15, 16]. Eindeutiger Handlungsbedarf (und auch Spielraum) besteht sicherlich bei dem Thema Fall‑/Entlassungsmanagement geriatrischer Patienten in der Notaufnahme. Angesichts des geringen Kontakts zu einer nachfolgenden (akut)geriatrischen Versorgung wäre eine verbesserte Nachversorgungsplanung zwingend mit der Einführung des Geriatrie-Checks oder ISAR zu verbinden, damit die Identifizierung nicht ins Leere läuft. Nicht zuletzt steht diese Entscheidung jedoch im Zusammenhang mit dem politischen Willen wie auch der Ausstattung der Notaufnahmen hinsichtlich dieser notwendigen Triage, welche inzwischen zwar gefordert, aber bislang nicht finanziell gefördert wird.

## Limitationen

Die Teilnehmer wurden überwiegend tagsüber an den Wochentagen rekrutiert, sodass eine höhere Behandlungsdringlichkeit infolge von Akuterkrankungen zu vermuten ist [5]. Es lässt sich nicht ausschließen, dass diese Akuterkrankungen Auswirkungen beispielsweise auf die kognitive Leistungsfähigkeit zeigten und die Ergebnisse beeinflussten. Zudem wurden pro Rekrutierungstag zwischen 4 und 6 Patienten in die vorliegende Studie eingeschlossen, jedoch im selben Zeitraum deutlich mehr Patienten über 70 Jahre in die Notaufnahme des Universitätsklinikums Ulm eingewiesen. Weiterhin handelt sich bei der rekrutierenden Station um eine rein internistische Notaufnahme und „chest pain unit“; chirurgische Patienten konnten nicht rekrutiert werden. Darüber hinaus konnten lediglich 146 Studienteilnehmer rekrutiert werden, während der ISAR auf Grundlage der Daten von 1673 Studienteilnehmern entwickelt und validiert wurde [14].

## Fazit für die Praxis

- Der Geriatrie-Check ist in der hier vorliegenden Studie ein dem Identification of Seniors at Risk (ISAR) vergleichbar valides Screeningverfahren mit einer hohen Sensitivität und hohen positiv-prädiktiven Werten, allerdings einer ebenso geringen Spezifität, insbesondere der prädiktiven Validität.
- Dies macht eine nachfolgende Validierung (sinnvoller 2‑stufiger Algorithmus) durch einen geriatrisch versierten Arzt/eine geriatrisch versierte Ärztin oder eine Pflegekraft notwendig.
- Nur etwa 15 % der vom Geriatrie-Check als geriatrisch identifizierten Patienten erhalten im Anschluss eine weitere akutgeriatrische Versorgung.
- Um zu überprüfen, ob sich der Test für eine sinnvolle Allokation von Ressourcen abschließend eignet, müssten weitere randomisierte Studien mit einer entsprechend größeren Studienpopulation durchgeführt werden.

## References

[1] Aminzadeh F, Dalziel WB (2002). Older adults in the emergency department. Ann Emerg Med.

[2] Baden-Württembergische Krankenhausgesellschaft e. V. (2013) Identifikation des geriatrischen Patienten. https://www.bwkg.de/index.php?eID=tx_nawsecuredl&u=0&g=0&t=1512489261&hash=32dcdb192c5010309506dd74e73578e87ad8f732&file=fileadmin/RELAUNCH/Downloads/04_Aufgaben-und-Services/BWKG_Arbeitshilfe_IdentifikationgerPatient.pdf. Zugegriffen: 4. Dez. 2017

[3] Biber R, Bail HJ, Sieber C (2013). Correlation between age, emergency department length of stay and hospital admission rate in emergency department patients aged ≥70 years. Gerontology.

[4] Carpenter CR, Shelton E, Fowler S (2015). Risk factors and screening instruments to predict adverse outcomes for undifferentiated older emergency department patients. Acad Emerg Med.

[5] Deschodt M, Devriendt E, Sabbe M (2015). Characteristics of older adults admitted to the emergency department (ED) and their risk factors for ED readmission based on comprehensive geriatric assessment. BMC Geriatr.

[6] Deutsche Gesellschaft für Geriatrie (DGG) Was ist Geriatrie? https://www.dggeriatrie.de/nachwuchs/91-was-ist-geriatrie.html. Zugegriffen: 6. Jan. 2020

[7] Hobert MA, Bernhard FP, Bettecken K (2019). Validierung des Geriatrie-Checks in einer Kohorte von stationären neurologischen Patienten. Z Gerontol Geriat.

[8] Hwang U, Carpenter C (2016). Assessing geriatric vulnerability for post emergency department adverse outcomes. Emerg Med J.

[9] Jorm AF (1999). The Informant Questionnaire on Cognitive Decline in the Elderly (IQCODE). Int Psychogeriatr.

[10] Karam G, Radden Z, Berall LE (2015). Efficacy of emergency department-based interventions designed to reduce repeat visits and other adverse outcomes for older patients after discharge. Geriatr Gerontol Int.

[11] Kondrup J, Rasmussen H, Hamberg O (2003). Nutritional risk screening (NRS 2002). Clin Nutr.

[12] Li Z, Jeon Y-H, Low L-F (2015). Validity of the geriatric depression scale and the collateral source version of the geriatric depression scale in nursing homes. Int Psychogeriatr.

[13] Mahoney FI, Barthel DW (1965). Functional Evaluation. Md State Med J.

[14] McCusker J, Bellavance F, Cardin S (1999). Detection of older people at increased risk of adverse health outcomes after an emergency visit. J Am Geriatr Soc.

[15] McCusker J, Jacobs P, Dendukuri N (2003). Cost-effectiveness of a brief two-stage emergency department intervention for high-risk elders. Ann Emerg Med.

[16] McCusker J, Dendukuri N, Tousignant P (2003). Rapid two-stage emergency department intervention for seniors. Acad Emerg Med.

[17] Ministerium für Arbeit und Sozialordnung, Familie, Frauen und Senioren (2014). Geriatriekonzept Baden-Württemberg 2014.

[18] Pötzsch O, Rößger F (2015) Bevölkerung Deutschlands bis 2060. https://www.destatis.de/DE/PresseService/Presse/Pressekonferenzen/2015/bevoelkerung/Pressebroschuere_Bevoelk2060.pdf?__blob=publicationFile. Zugegriffen: 4. Dez. 2017

[19] Qureshi KN, Hodkinson HM (1974). Evaluation of a ten-question mental test in the institutionalized elderly. Age Ageing.

[20] Ranhoff AH, Laake K (1993). The Barthel ADL index. Age Ageing.

[21] Salvi F, Morichi V, Grilli A (2012). Screening for frailty in elderly emergency department patients by using the Identification of Seniors At Risk (ISAR). J Nutr Health Aging.

[22] Singler K, Christ M, Sieber C (2011). Geriatrische Patienten in Notaufnahme und Intensivmedizin. Internist.

[23] Singler K, Dormann H, Dodt C (2016). Der geriatrische Patient in der Notaufnahme. Notfall Rettungsmed.

[24] Thiem U, Greuel HW, Reingräber A (2012). Positionspapier zur Identifizierung geriatrischer Patienten in Notaufnahmen in Deutschland. Z Gerontol Geriat.

[25] Yao J-L, Fang J, Lou Q-Q (2015). A systematic review of the identification of seniors at risk (ISAR) tool for the prediction of adverse outcome in elderly patients seen in the emergency department. Int J Clin Exp Med.

[26] Yesavage JA, Brink TL, Rose TL (1982). Development and validation of a geriatric depression screening scale. J Psychiatr Res.

